# Present and Future of Phase-Selectively Disordered Blue TiO_2_ for Energy and Society Sustainability

**DOI:** 10.1007/s40820-020-00569-0

**Published:** 2021-01-04

**Authors:** Yongguang Luo, Hyoyoung Lee

**Affiliations:** 1 Center for Integrated Nanostructure Physics (CINAP), Institute for Basic Science (IBS), 2066 Seoburo, Jangan-gu, Suwon, 16419 Republic of Korea; 2 Department of Chemistry, Sungkyunkwan University, 2066 Seoburo, Jangan-gu, Suwon, 16419 Republic of Korea; 3 Creative Research Institute (CRI), Sungkyunkwan University, 2066 Seoburo, Jangan-gu, Suwon, 16419 Republic of Korea; 4 Department of Biophysics, Sungkyunkwan University, 2066 Seoburo, Jangan-gu, Suwon, 16419 Republic of Korea

**Keywords:** Blue TiO_2_ (BTO), Phase-selective disordering, Visible-light-driven photocatalyst, Charge separation, Energy and society sustainability

## Abstract

Titanium dioxide (TiO_2_) has garnered attention for its promising photocatalytic activity, energy storage capability, low cost, high chemical stability, and nontoxicity. However, conventional TiO_2_ has low energy harvesting efficiency and charge separation ability, though the recently developed black TiO_2_ formed under high temperature or pressure has achieved elevated performance. The phase-selectively ordered/disordered blue TiO_2_ (BTO), which has visible-light absorption and efficient exciton disassociation, can be formed under normal pressure and temperature (NPT) conditions. This perspective article first discusses TiO_2_ materials development milestones and insights of the BTO structure and construction mechanism. Then, current applications of BTO and potential extensions are summarized and suggested, respectively, including hydrogen (H_2_) production, carbon dioxide (CO_2_) and nitrogen (N_2_) reduction, pollutant degradation, microbial disinfection, and energy storage. Last, future research prospects are proposed for BTO to advance energy and environmental sustainability by exploiting different strategies and aspects. The unique NPT-synthesized BTO can offer more societally beneficial applications if its potential is fully explored by the research community.

## Highlights

- Milestones of TiO_2_ development and invention of phase-selectively ordered/disordered blue TiO_2_ (BTO) is in-depth illustrated.
- The explored and potential applications of BTO are reviewed and proposed thoroughly.
- The forthcoming flourishing research trends based on account of BTO are suggested.

## Graphical abstract

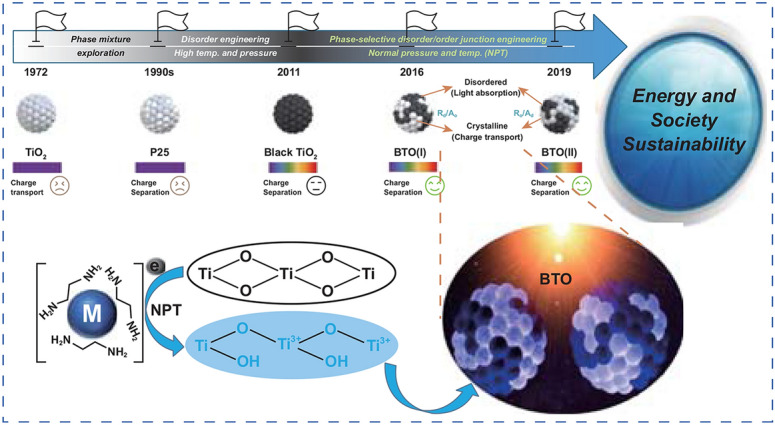

## Introduction

Modern society has achieved great science and technology explosion to date but faces severe energy demands and environmental concerns to realize sustainable development. One crucial issue in the twenty-first century is finding ways to convert and store renewable energy efficaciously while tackling climate change and environmental pollution caused by unsustainable human activity. In that context, titanium dioxide (TiO_2_) has received a lot of attention for its photocatalytic activity, energy storage capability, low cost, high chemical stability, and nontoxicity. The initial discovery of the photocatalytic potential of TiO_2_ dates back to the end of the 1920s [1]. Researchers observed that aniline dyes faded and fabrics degraded in the presence of TiO_2_, oxygen gas (O_2_), and ultraviolet (UV) light. However, the academic community did not show strong scientific enthusiasm about the phenomenon at that time due to a lack of interest in renewable energy and environmental stewardship.

That began to change after Fujishima and Honda reported the discovery of water photoelectrolysis into hydrogen (H_2_) by rutile TiO_2_ under UV irradiation in 1969 [2]. The Honda–Fujishima water splitting finding was refined and called “natural photosynthesis” by *Nature* in 1972 [3]. Now, TiO_2_ is one of the most promising photocatalyst materials. Its valence band (VB) and conduction band (CB) positions offer more diverse catalysis reaction potential than available with many other transition metal oxides and dichalcogenides [4]. Furthermore, the heterogeneous photocatalysis of TiO_2_ enables smoother industrial recycling than is available for homogeneous photocatalysts.

TiO_2_ has three main polymorphs, anatase, rutile, and brookite. The anatase and rutile phases of TiO_2_ are the most frequently studied and synthesized in laboratories and industry, whereas brookite, as a natural phase, is rarely investigated as a photocatalyst due to difficulties in synthesizing it [5]. The anatase phase is reported to have higher photocatalytic performance than the rutile phase because it has better bulk charge transportation and a smaller recombination portion of the exciton [6, 7]. After several decades of developments, various TiO_2_ synthesis approaches have been established through gas-phase reactions, solution-based methods, and alcoholysis from titanium tetrachloride (TiCl_4_) [8], titanium oxysulfate (TiOSO_4_) [9], and Ti(OC_4_H_9_)_4_ [10]. To date, one of the famous TiO_2_ photocatalyst products is commercial Degussa P-25 (P25) (Degussa Co., Ltd), which has been frequently applied as a benchmark photocatalyst [11, 12]. P25 TiO_2_ contains a unique hybrid of anatase and rutile phases in a roughly 3:1 ratio and exhibits good performance in many photocatalytic systems [13]. TiO_2_ is also used in other industries: energy (energy production and storage), environment (degrading pollution in the air, wastewater, and indoors), human health and food (antibacterial, antivirus sterilization), cosmetics (sunscreen against UVA and UVB), and self-cleaning and antifogging products [14, 15]. The antivirus potential of TiO_2_ will certainly draw attention during the struggle with the COVID-19 coronavirus pandemic [16].

TiO_2_ still faces several hindrances to its photocatalytic performance. First, pristine TiO_2_ can absorb sunlight only in the UV region (5%) due to its large electronic bandgaps (anatase, 3.2 eV; rutile 3.0 eV), which results in extremely low photocatalysis quantum efficiency that fails to meet the needs of industrial applications. Second, the separated charges (electrons and holes) formed after photoexcitation of a photocatalyst can recombine and disappear, giving subsequent photoluminescence. This exciton recombination process reduces the number of active electrons and holes on the photocatalyst surface, which is detrimental to any photocatalysis reaction. Therefore, research is needed to boost light absorption efficiency and block charge recombination to maintain a high exciton dissociation capability. Many attempts have been made to attain a broader range of light absorption by using non-metallic elements (C, N, and S) [17] and transition metal doping [18, 19] to tune the TiO_2_ electronic structure. However, only a few researchers have tried to develop advanced TiO_2_ by targeting both visible-light absorption and high charge separation efficiency. Those efforts produced “Black TiO_2_” [20] and “Blue TiO_2_” [21] in 2011 and 2016, respectively. In addition, the phase-selectively disordered blue TiO_2_ (BTO) offers high H_2_ generation performance through its three unique phase–interface configurations.

Next, we summarize several historical milestones in the development of TiO_2_ materials and then systematically illustrate the development logic and discovery of BTO, including its mild synthesis conditions, robust reducing agent design, and phase-selective disordering. The phase selectivity of BTO results from its unique structure, which we disclose on the crystalline dimension level, and the reduction power of alkali metal amines. Its ordered-disordered phase junctions, type II band alignment structure, and a surface rich in hydroxyl groups explain the high exciton dissociation efficiency, visible-light absorption, and superior photocatalysis of BTO. Particularly, we further present the exploratory attempts of BTO in various energy and environmental aspects. Finally, we suggest future research avenues to explore the potential of BTO further.

## Milestones in TiO_2_ and the Development of BTO

**Fig. 1:**
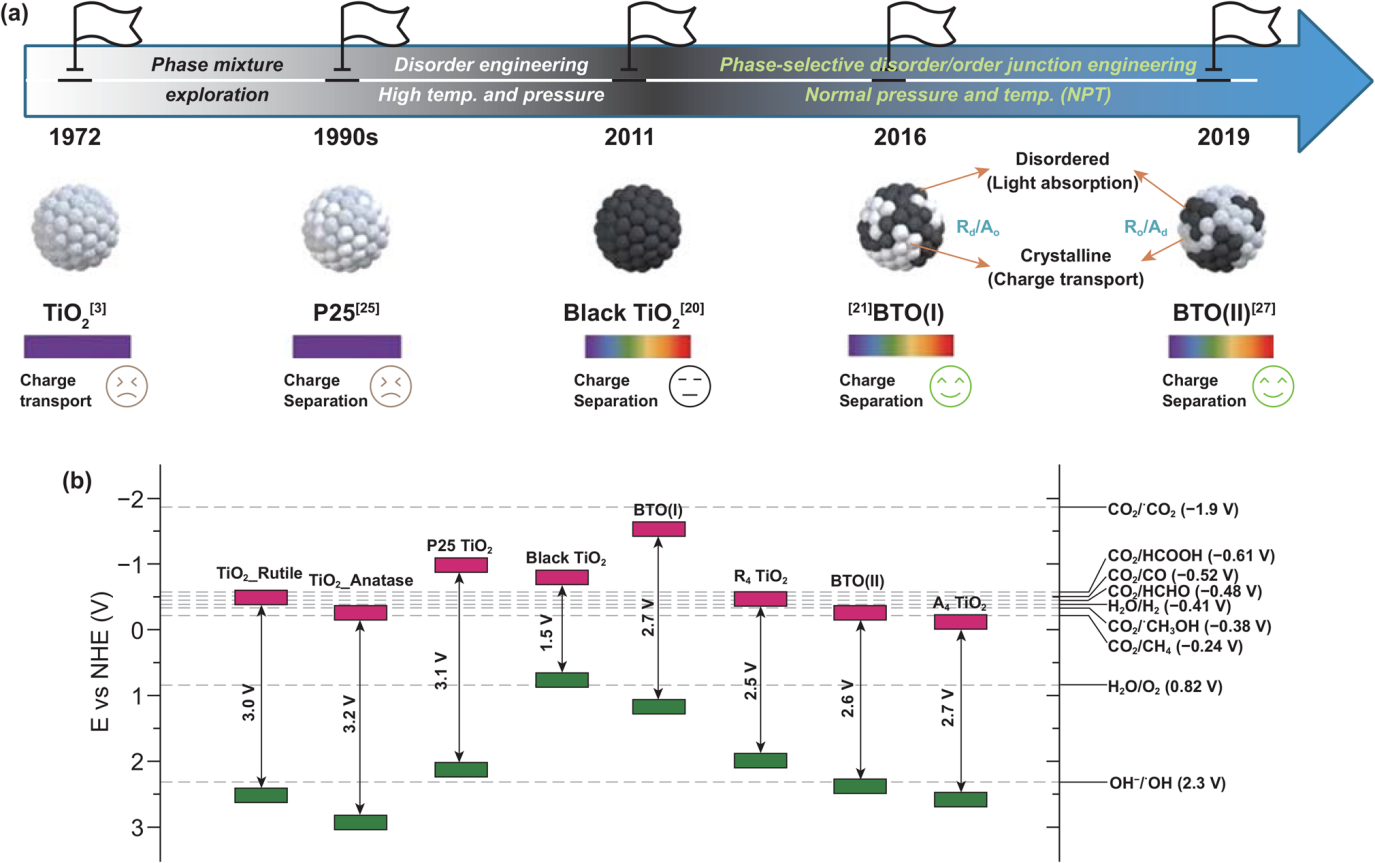
**a** Milestones in TiO_2_ material development and **b** the corresponding band structure of each typical TiO_2_ configuration [3, 20, 21, 25, 28, 32]. The TiO_2_ nanoparticles illustration figures in (**a**) are adapted with the permission from Ref. [28]. Copyright (2019) American Chemical Society

The research community has long worked to exploit the energy efficiency and activity of TiO_2_ in versatile applications. Events of considerable significance in the recent history of TiO_2_ development are shown in Fig. 1a. The first fundamental finding in TiO_2_ photocatalysis for energy conversion was a report of water electrochemical photolysis which generates the absolute clean energy gas, H_2_, using a rutile TiO_2_ semiconductor electrode in 1972 by Fujishima and Honda et al. However, the applied rutile TiO_2_ has lower charge transportability than anatase TiO_2_, even though it has better light absorption efficiency to generate more charges. The underlying reasons that anatase has better charge transportation than the rutile phase are the higher VB maximum energy level of the anatase phase (Fig. 1b) [7], its preferred crystalline surface orientation [22], and its longer exciton (electron and hole pair) lifetime [23]. To use the advantages of both the anatase and rutile phases, the commercially available phase-mixed P25 TiO_2_ has been widely used as a standard photocatalyst. It has been proved a better activity than the single-phase TiO_2_ since the 1990s [13, 24, 25]. Extending the TiO_2_ light absorption range was the main challenge after the development of P25. In 2011, Xiaobo Chen et al. found that TiO_2_ phase disorder engineering through hydrogenation enhanced its light absorption capability into the visible and infrared ranges [20]. That hydrogenated black TiO_2_ mostly answered concerns about the photocatalysis energy efficiency of TiO_2_. Moreover, the photocatalytic activity of black TiO_2_ is boosted by suppressing exciton recombination through the middle VB by means of localized holes generated in the disordered surface. During approximately four decades of research, TiO_2_ material development has produced decent UV–visible-light absorption, acceptable photocatalytic activity, and reasonable charge generation. Nevertheless, TiO_2_ still requires more development to be practical for energy production and photocatalysis applications. After thoroughly investigating the recent achievements in black TiO_2_ material design, we found that most black TiO_2_ synthesis approaches require high temperatures (400–900 °C) or a high H_2_ atmospheric pressure (20–70 bar) [26]. Furthermore, the black TiO_2_ core/shell structure produces a back reaction that diminishes its photocatalytic power because of its sole surface reaction interface. The issues that remain to be addressed since the development of black TiO_2_ are developing an industrially suitable manufacturing process with mild conditions and further strengthening exciton dissociation and catalytic reaction efficiency.

## NPT Synthesis of BTO and Its Phase-selective Specialty

The high-temperature and H_2_ atmospheric pressure synthesis conditions of most black TiO_2_ are energy-intensive and potentially explosive, an unfavorable manufacturing choice in both laboratories and industry. Therefore, it is essential to find a suitably, potent reducing agent or system. Birch reduction agents, an alkali metal in liquid ammonia, can reduce arenes into cyclohexadiene rather than cyclohexane [27]. Among the Birch reduction processes, the electride salts that form by mixing an alkali metal (M) and ammonia (NH_3_) as [M(NH3)_x_]^+^ e^−^ have strong reducing power. Therefore, we supposed that producing such vital electride salts as a reduction species would contribute to TiO_2_ reduction. We found that a lithium ethylenediamine (Li-EDA) solution reduced the rutile phase of P25 TiO_2_ while keeping the anatase phase intact, which resulted in a unique blue TiO_2_ product (BTO(I)) [21].

**Fig. 2:**
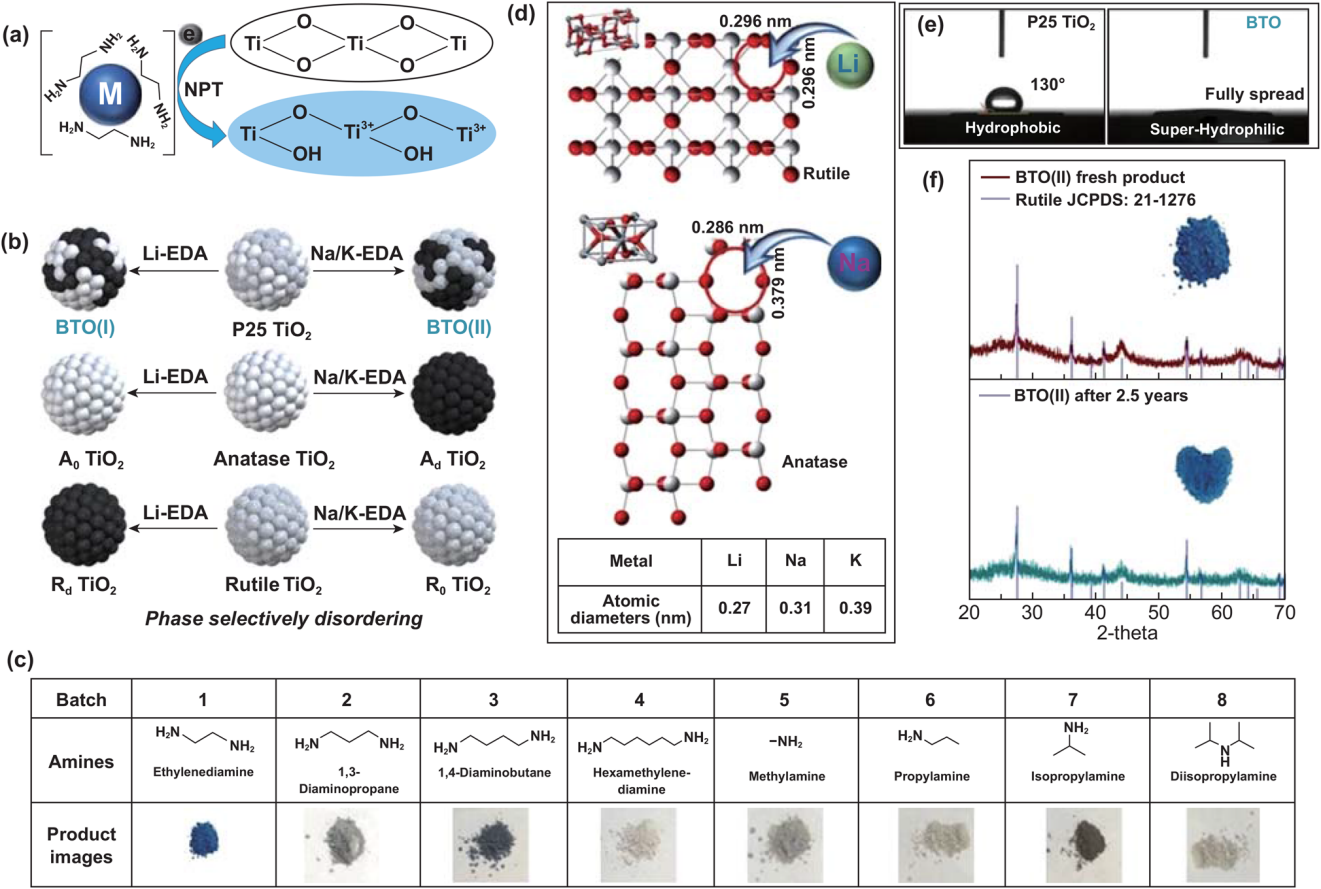
NPT synthesis of BTO and its phase selectivity. **a** M-EDA electrides reduce pristine TiO_2_ to BTO under NPT conditions. **b** Different starting TiO_2_ phases are selectively reduced/disordered by M-EDA solutions. **c** Amine solvent investigation to synthesize BTO. Adapted with permission from Ref. [28]. **d** Proposed mechanism for the BTO phase-selective phenomenon. Adapted with permission from Ref. [28, 29]. **e** Water contact angle measurements (SEO PHX300) of the original P25 TiO_2_ and the phase-selectively reduced BTO. **f** Structure and appearance stability characterization by X-ray powder diffraction (SmartLab JD3643N) and digital photo images

The superior photocatalysis performance of BTO stimulated us to investigate the origin of its phase selectivity further. As shown in Fig. 2a, the free electron of M-EDA electrides can attack the firm Ti–O bond and produce a reduced Ti^3+^ state. The evidence for Ti^3+^ and oxygen vacancy (OV) were provided by X-ray photoelectron spectroscopy and electron paramagnetic resonance in our previous reports from 2015 to 2019, respectively. Besides, the reduced TiO_2_ is generally presented in a disordered amorphous physical state with a black appearance. The blue color of BTO is caused by the coexistence of an ordered crystalline anatase (A_o_) phase and a disordered amorphous rutile (R_d_) phase. The successful reduction of TiO_2_ by a Li-EDA solution in normal pressure and temperature (NPT) conditions indicates its mighty reducing power. It successfully replaced the high pressure and temperature hydrogenation reduction approach.

After successfully preparing ordered anatase (A_o_)/disordered rutile (R_d_) TiO_2_ from P25 with the Li-EDA solution under NPT conditions, we set out to design disordered anatase (A_d_)/ordered rutile (R_o_) TiO_2_ from P25. With the curiosity of other alkali metal EDA reduction phenomena, we applied Na and K EDA solutions to reduce the P25. Interestingly, the Na/K-EDA solution selectively reduced the P25 TiO_2_ reverse from the Li-EDA. Figure 2b shows that P25 TiO_2_ turns to R_d_/A_o_ (BTO(I)) through Li-EDA reduction and R_o_/A_d_ (BTO(II)) through Na/K-EDA reduction. Furthermore, the anatase and rutile phases TiO_2_ were individually treated by Li-EDA and Na/K-EDA, respectively. The pure white rutile TiO_2_ becomes black R_d_ TiO_2_ in a Li-EDA environment, and the anatase single-crystalline form becomes black or gray A_d_ TiO_2_ after the Na/K-EDA reduction. Based on that initial finding, which we were the first to report, the TiO_2_ architecture can be widely enriched to extend its potential applications. Because of their blended ordered and disordered phase structure, BTO(I) and BTO(II) have high potential as photocatalysts with effective heterojunctions and visible-light absorption. In addition, the M-EDA-reduced R_d_ and A_d_ can be used to anchor hybrid material systems and maintain a steady structure through covalent combinations.

To investigate the best amines for dissolving alkali metals and reducing TiO_2_, we selected various liquid amine derivatives, including monoamines with different alkyl chain lengths (Numb. 1–4 in Fig. 2c) and diamines with diverse alkyl chain lengths and positions (Numb. 5–8 in Fig. 2c). The various M-amine solutions produced diverse forms from the P25 that were colored from blue to gray. Among them, the shortest alkyl chain diamine solution, Na-EDA, exhibited the best reduction results, producing a deep blue color and entirely vanished anatase crystalline phase, as shown in the detailed XRD characterization in Ref. [28]. EDA's effects result from its effective diamine structure and higher polarity than the long alkyl chain amines, which contribute to its high alkali metal solubility. This newly developed, powerful reducing system (M-EDA) can be readily extended to the reduction of other metal oxides or metal sulfides and defect design objectives.

Next, we examined the crystallography of anatase and rutile TiO_2_ at the atomic level to find the origins of the phase selectivity. Beginning with facet information about the rutile (110) and anatase (101) phases [28–30], we found the gap distance in the unit lattice to be around 2.96 Å × 2.96 Å for rutile (110) and 2.86 × 3.79 Å^2^ for anatase (101), as shown in Fig. 2d. The diameters of Li, Na, and K atoms in the EDA environment are 2.7, 3.1, and 3.9 Å, respectively, as shown in the inserted table in Fig. 2d. Clues about phase selectivity can be drawn from that lattice and atomic size information. Na and K, which are larger than Li, are relatively close to the anatase (101) lattice unit dimensions but more massive than the rutile (110) lattice gaps. Therefore, Na and K can attack Ti–O-Ti bonds in the anatase phase and break the anatase crystalline into a disordered state. On the other hand, Li atoms can effectively attach to the rutile (110) lattice units, rather than the wider lattice spaces of the anatase (101), and thus successfully reduce only the rutile TiO_2_ phase. In that way, the intrinsic adaptability of Li-EDA to the rutile phase and Na/K-EDA to the anatase phase determine the selectivity of the disordering results.

Furthermore, the M-EDA treatment process is easy to scale up and highly repeatable through the alkali metal stepwise feeding. Using a hydrophilic material is necessary to provide good interfacial contact in many photocatalysis and other real-world applications. As shown in Fig. 2e, a water drop fully spreads on the BTO film, which indicates that BTO is more hydrophilic than pristine P25 TiO_2_. The excellent hydrophilicity of BTO originates from the enriched surface hydroxyl (OH) groups that appear after the M-EDA reduction. Pristine P25 TiO_2_ has a hydrophobic surface, with a 130° water contact angle, due to the absence of hydrophilic functional groups on its intact TiO_2_ surface. Material stability is another concern for practical applications. BTO has maintained its original disordered/ordered structure and appearance for almost 2.5 years under ambient conditions, as represented in Fig. 2f. Thus, BTO has many advantages, from low-cost production to high potential for many practical applications.

## Explored and Potential Applications of BTO

BTO exhibits strong visible-light (380–740 nm) absorption ability with a narrow optical bandgap (Fig. 1b), efficient photoinduced exciton disassociation with a heterojunction structure [21], and excellent hydrophilicity and stability (Fig. 2e, f). Our group has applied BTO to promote green energy and social sustainability in the field of hydrogenation [21], algae elimination from aquatic ecosystems [31], carbon dioxide (CO_2_) reduction [28, 32], and visible-light-driven organic synthesis (C–H arylation) [33]. Next, we describe those BTO applications and then propose strategies and directions for further designs and applications of BTO.

### Explored Photocatalytic Aspects of BTO

**Fig. 3:**
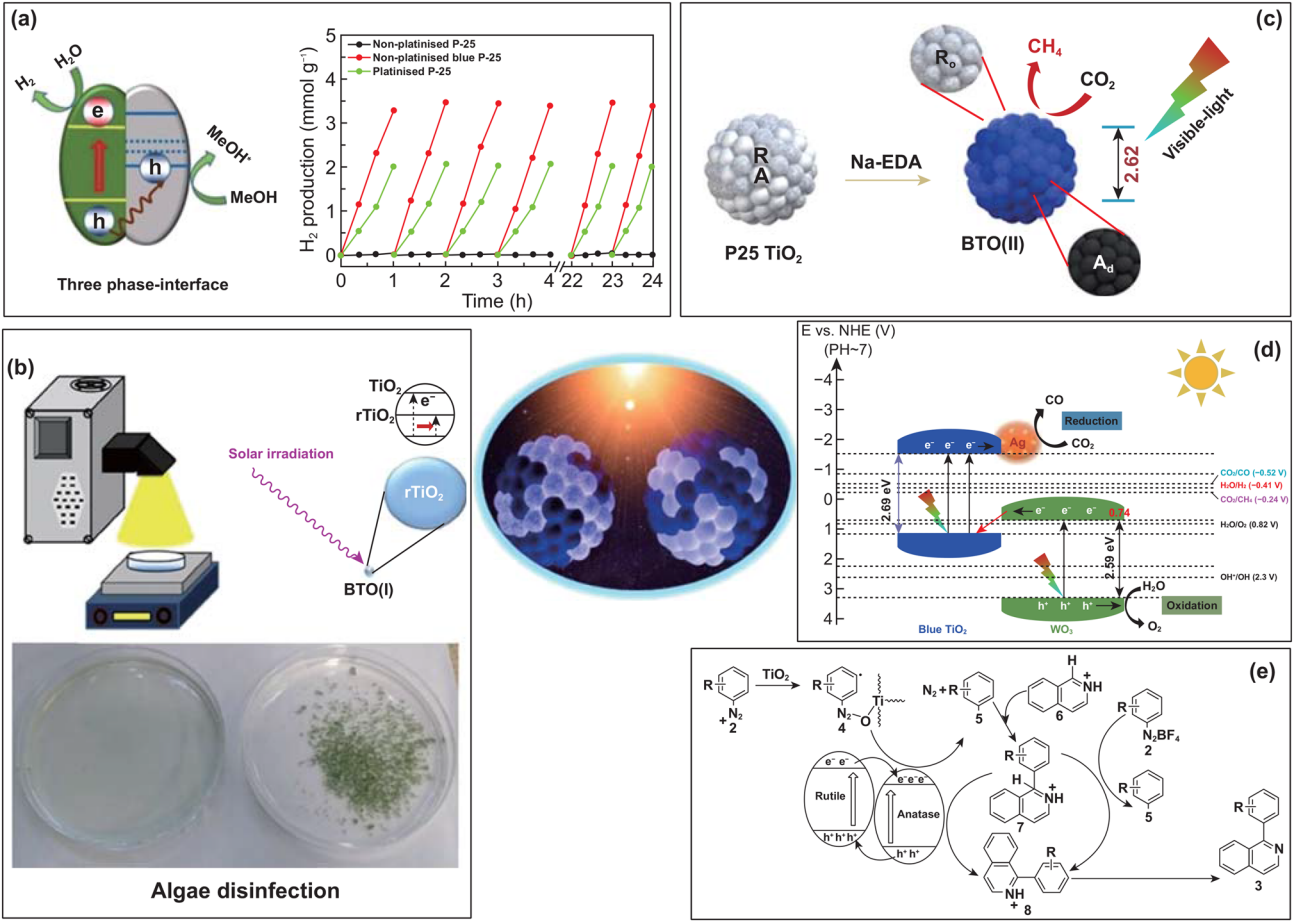
Explored photocatalytic applications for BTO. **a** Unique three-phase-interface BTO(I) robust H_2_ photogeneration from water. Adapted with permission from Ref. [21]. **b** Efficient Chlamydomonas green algae disinfection by BTO(I) under solar irradiation. Adapted with permission from Ref. [31]. **c** Visible-light-driven CO_2_ reduction (CO_2_RR) to CH_4_ by BTO(II). Adapted with permission from Ref. [28]. **d** BTO(I)/WO_3_-Ag combination with a Z-scheme band structure for high-selectivity CO_2_RR to CO. Adapted with permission from Ref. [32]. **e** BTO(I) photocatalytic activity in C–H arylation organic synthesis. Adapted with permission from Ref. [33]

Hydrogen has been deemed a perfect blue energy source that could solve the energy crisis in the twenty-first century. For example, its heating value (141.72 MJ kg^−1^) is three times higher than gasoline (46.4 MJ kg^−1^) [34], and it is an extremely abundant material that produces zero pollution and has reproducible capabilities through the water. Solar-driven photohydrogenation has received much attention because of its high sustainability. BTO, as a typical semiconductor material, can be used as a robust hydrogenation photocatalyst and has shown a remarkable performance enhancement over P25 and most other reported TiO_2_ materials [21]. The extended light absorption spectrum of BTO covers all solar illumination, which maximizes the quantum efficiency of its photocatalysis process. However, it is not enough to have a favorable light-harvesting ability; a desirable hydrogenation photocatalyst must also produce effective charge separation through a specific structure designation. As shown in Fig. 3a, BTO retains discrete catalytic redox reaction sites for the reduction of water to hydrogen and methanol sacrificial agent oxidation. The right-side gray R_d_ is responsible for absorbing enough light irradiation and generating the photoinduced electron and holes. Afterward, the adjacent A_o_ accepts electrons to trigger water splitting. Compared with the conventional core–shell structure of black TiO_2_, BTO eliminates the need for electrons to migrate from the core to the interface of the shell and water. Therefore, it greatly reduces the potential for charge recombination. Furthermore, the type II band alignment configuration of BTO assists in exciton dissociation and keeping the effective charges. The open ordered/disordered structure of BTO realized a superior H_2_ production rate of 13.89 mmol h^−1^ g^−1^ with 0.5 wt% Pt and 3.46 mmol h^−1^ g^−1^ without the Pt co-catalyst.

Algae blooms happen regionally in various brine and river systems, mainly due to water eutrophication induced by human activities, and they damage public health and the social economy [35]. Severe overgrowth of algae can kill aquatic creatures by consuming the limited oxygen dissolved in the water. TiO_2_, as a typical photocatalyst, can generate reactive oxygen species (ROS), which mainly consist of a hydroxyl radical (·OH) and superoxide anion radicals (·O_2_^−^), through photo irradiated hot carriers that attack water molecules. The ROS then remove algae. However, conventional TiO_2_ has low efficiency in generating sufficient ROS for algae elimination. Based on our previously obtained photocatalytic hydrogenation experience, we applied BTO to remove Chlamydomonas green algae (Fig. 3b) [31]. We expected that the powerfully wide range of light absorption and effective charge separation properties of BTO would produce an efficient ROS amount. The algae removal test was conducted under both UV and solar light with various types of TiO_2_. The BTO wiped out all the algae cells within 2–2.5 h, which was the most rapid among the kinds of TiO_2_ tested. Thus, BTO has meaningful roles to play in realizing a sustainable society.

The photoreduction of CO_2_ into chemical fuels under solar or visible light is supposed to be an excellent way to target both energy and environmental concerns. This so-called artificial photosynthesis strategy has been under study for a while, but desirable conversion selectivity and production yield are still lacking [36]. Moreover, it is quite hard to crack the C = O bonds in the CO_2_ molecule because the dissociation energy demand is high (around 750 kJ mol^−1^) [37]. The ideal photocatalyst for the CO_2_ reduction reaction (CO_2_RR) needs a specific configuration with an optical band position (especially the CB) that is close to the CO_2_ reduction potential, such as the − 0.24 V_NHE_ of CO_2_ to CH_4_ or the − 0.52 V_NHE_ of CO_2_/CO, and also efficient charge separation with good electron transport. BTO has those structures, so we conducted CO_2_ reduction experiments using BTO(II) under visible light [28] and BTO hybrid materials (BTO(I)/WO_3_-Ag) under solar light [32]. The BTO(II) reached unprecedented CH_4_ production levels (3.98 µmol g^−1^ h^−1^), with the highest yield among all the metal (Pt, Ru, W, and Ag)-doped P25 TiO_2_ materials tested. The evident CO_2_RR ability was conferred by the excellent match between the CB position of BTO(II) (− 0.24 V_NHE_) and the CO_2_ to CH_4_ potential and the efficient visible-light absorption by the A_d_ with rapid charge-carrier disassociation (Fig. 3c). Even though BTO(II) offered excellent CO_2_RR performance, another critical issue for CO_2_RR, product selectivity, also has to be addressed. Consequently, we designed and constructed BTO(I)/WO_3_-Ag, a combination material intended to build a particular *Z*-scheme band structure, as presented in Fig. 3d. The assembled *Z*-scheme band alignment can maximize the effective potential between a high CB and low VB and then strengthen the catalytic redox power. Notably, the CB position (− 1.55 V_NHE_) of BTO(I) is close to the CO_2_ to CO potential (Fig. 1b), which contributes to CO production, and higher than the CB of WO_3_ (0.74 V_NHE_) used to construct the *Z*-scheme band alignment. In addition, the low difference between the VB of BTO(I) (1.14 V_NHE_) and the CB of WO_3_ facilitates the flow of excited WO_3_ electrons to BTO(I), thereby reinforcing the number of effective hot electrons. The decorated Ag nanoparticles serve as an electron reservoir that can initiate photoelectron production by means of the localized surface plasmon resonance effect and further enhance visible-light absorption. When tested, this BTO based *Z*-scheme composite produced absolute CO selective-production of 1166.7 µmol g^−1^ h^−1^ at the excellent photocatalytic electron reaction pace of 2333.4 µmol g^−1^ h^−1^. All in all, BTO showed vigorous CO_2_RR strength in producing CH_4_ or CO with high output and selectivity, which means it can be an attractive way to tackle global warming and energy deficiency together.

Light-driven chemical synthesis is also an essential field that requires promising photocatalysts to boost synthesizing efficiency [38]. C–H arylation for organic synthesis was chosen as a typical study case to show the photocatalytic activity of BTO (Fig. 3e) [33]. The phase-mixed BTO absorbs light in the visible range through its Ti^3+^ defect-rich disordered state. It maintains good adsorptivity of an organic reactant and charges separation via its ordered crystalline phase. First, a charge transfer complex (**4**) formed on the A_o_ site of BTO(I) from the aryl diazonium compound (**2**). Then, under visible-light irradiation, photogenerated electrons flowed to the anatase CB due to the type II band alignment and efficiently separated from the holes. An aryl intermediate radical (**5**) was produced after the single electron transfer process from A_o_ to (**4**). As arylation proceeded, after initiation by aryl radical (**5**), the resulting radical (**7**) intermediate was oxidized by the hot hole carrier from the A_o_ VB and gave the desired product (**3**) after deprotonation of the product (**8**). Moreover, BTO offers high reusability through direct filtration, and it maintained consistent yield (63%) performance when five batches were examined under sixfold scaled-up conditions. This application of BTO to photocatalytic chemical synthesis will enrich the role of TiO_2_ in industrial chemical synthesis and contribute to further product cost reductions.

### Potential Application and Design Commentary of BTO

**Fig. 4:**
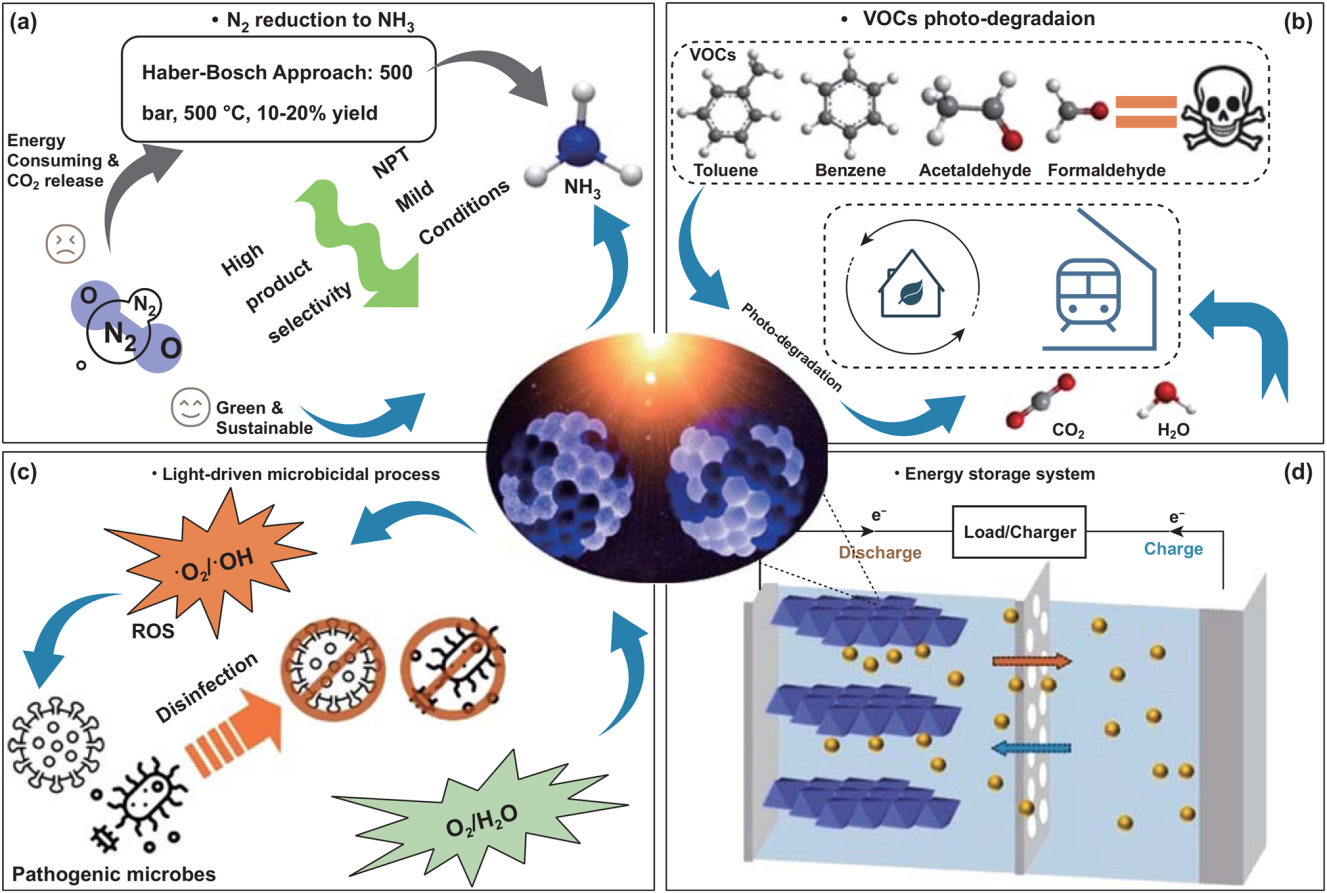
Potential applications and design commentary of BTO. **a** Photo-driven N_2_ reduction to NH_3_ to replace the conventional Haber–Bosch approach. **b** Photodegradation of volatile organic compounds (VOCs), especially in indoor atmospheres. **c** Adapting to visible-light-induced microbicidal processes. **d** Exploring BTO as electrode material in an energy storage system by taking advantage of its electro-conducting Ti^3+^ species, oxygen vacancy, and stability

Currently, ammonia (NH_3_) synthesis from nitrogen gas (N_2_) is an essential approach to supplying nitrogen to plants and humans by industry manufacturing. The Haber–Bosch process for NH_3_ synthesis (N_2_ + H_2_ → NH_3_), which has been used in industry for more than a century, urgently needs to be replaced due to its high consumption of fossil fuels, which results in enormous greenhouse gas (CO_2_) emissions and extremely harsh operating conditions (400–500 °C, 100–200 bar with an iron-based catalyst) [39]. Therefore, photocatalysis nitrogen fixation that can use sustainable solar energy and eliminate CO_2_ emissions has attracted growing attention. However, it remains challenging to design an efficient photocatalyst to convert N_2_ to NH_3_ under NPT conditions with a high production rate and clear mechanism [40]. It has been reported since 1977 that TiO_2_ generated NH_3_ and other gasses under UV irradiation with an N_2_ source, but the process offered minimal yields and low selectivity [41]. After several decades of progress, the yields from TiO_2_-driven photosynthesis of NH_3_ have been enhanced by hundreds-fold [40]. However, most related studies still apply only UV light because of the narrow light absorption region of conventional TiO_2_, which hinders the application range and produces low solar coulombic efficiency. As illustrated in Fig. 4a, by tracking the advanced TiO_2_ photocatalyst design milestones, it has high credits to investigate the N_2_ fixation to NH_3_ by taking phase-selective disordering and visible-light harvesting advantages of BTO for targeting maximized NH_3_ production yield and selectively under mild NPT conditions.

In recent years, volatile organic compounds (VOCs), which vaporize easily at room temperature, have become major hazardous pollutants in the air through speedy industrialization and urbanization. Some studies show that indoor atmospheres can have 2–10 times more VOCs than outdoor environments [42]. Therefore, VOCs' health concerns, such as cancer, headaches, and dizziness, are serious among people who spend most of their time in buildings or enclosed spaces. Among the various VOCs, toluene, benzene, and aldehydes (formaldehyde and acetaldehyde) are the most common and toxic species [43]. Photodegradation of VOCs is inevitably regarded as the best and most economical choice for dealing with VOCs in the air. The carbon–carbon bonding and carbonyl groups in VOC molecules are comparatively stable, requiring sufficiently hot carriers from a powerful photocatalyst to be decomposed. BTO is expected to actively cause full VOC degradation into CO_2_ and H_2_O by effectively generating photoinduced charges and inhibiting exciton recombination under solar and indoor LED lamplight (Fig. 4b). Additionally, the hydroxyl-rich character of the disordered portion of BTO can specifically support the covalent coating and binding process on various substrates and objects (such as air conditioner filters, indoor walls, and subway carriages) and thereby provide versatile application choices.

Microbial pathogens, which include various bacteria and viruses, are major health concerns to humans worldwide. They occasionally cause serious infectious disease pandemics, such as those caused by the novel coronavirus (COVID-19), severe acute respiratory syndrome coronavirus (SARS), swine influenza virus (H1N1), and Middle-East respiratory syndrome coronavirus (MERS) [16]. For the sake of human health, society needs effective microbial disinfection systems with enough versatility to attack airborne, waterborne, and foodborne pathogenic species. Practically, various microbicidal processes have already been adopted, such as UV disinfection, antibiotic sterilization, thermal treatments, and nanofiltration. However, the current approaches possess significant limitations; for instance, some microbes have already evolved antibiotic or UV resistance [44], and thermal, and filtration operations can cause energy exhaustion and are incompatible in many spaces. The microbial pathogens inactivation by TiO_2_ photocatalyst can trace to 1994 after the Sjogren et al. finds the inactivation ability to bacteriophage MS2 on TiO_2_ [45]. Besides, TiO_2_ could be a good option for microorganism disinfection that is low cost, requires minimal energy consumption, and is harmless and eco-friendly [46, 47]. In the TiO_2_ photocatalysis microorganism disinfection process, the ROS generated from photocatalytic processes after light irradiation plays the major roles [16]. To further boost the microbial pathogens inactivation performance of TiO_2_, we need to strengthen the producing amount of ROS species. In the authors’ group previous reports, BTO can generate a higher amount of ROS species under UV, visible or solar light illumination, which is represented by the higher peak intensity of BTO than pristine TiO_2_ in electron paramagnetic resonance analysis [31, 48]. Therefore, BTO could act as a broad-spectrum antimicrobial agent and outperform pristine TiO_2_ by generating sufficient antibacterial and viricidal ROS at different band positions, as depicted in Fig. 4c. Harmful bacteria and viruses in living spaces could be effectively deactivated under mild conditions by using BTO and solar or visible light.

Because TiO_2_ has superior stability, high safety, and good economic value, it has been investigated and considered as an anode or cathode candidate in various ion battery systems, including single-valent alkali-ion batteries (LIBs, SIBs, and KIBs) [49], multivalent magnesium ion batteries (MIBs) [50] and aluminum ion batteries (AIBs) [51]. Also, researchers have noticed that Ti^3+^ self-doped black anatase TiO_2_ has better rate capability than pristine white anatase in LIBs [52], and the associated OV of black TiO_2_ resulted in high-performance magnesium ion (Mg^2+^) storage [50]. Nevertheless, the synthesis of black TiO_2_ requires a high-temperature reduction process, and their black TiO_2_ products remain in a majority crystalline phase and only acquire a small portion Ti^3+^; even the OV and Ti^3+^ was suggested as main contributions to the advances. Therefore, we propose BTO (including the A_d_ and R_d_ synthesized by M-EDA) as an encouraging candidate for battery system electrodes (Fig. 4d). Our M-EDA reduction approach, along with the production of BTO under NPT conditions, can almost completely disorder anatase (Na-EDA) and rutile (Li-EDA) TiO_2_ and deliver sufficient OVs and electro-conducting Ti^3+^ species to enhance energy storage performance.

## Prospects and Summary

**Fig. 5:**
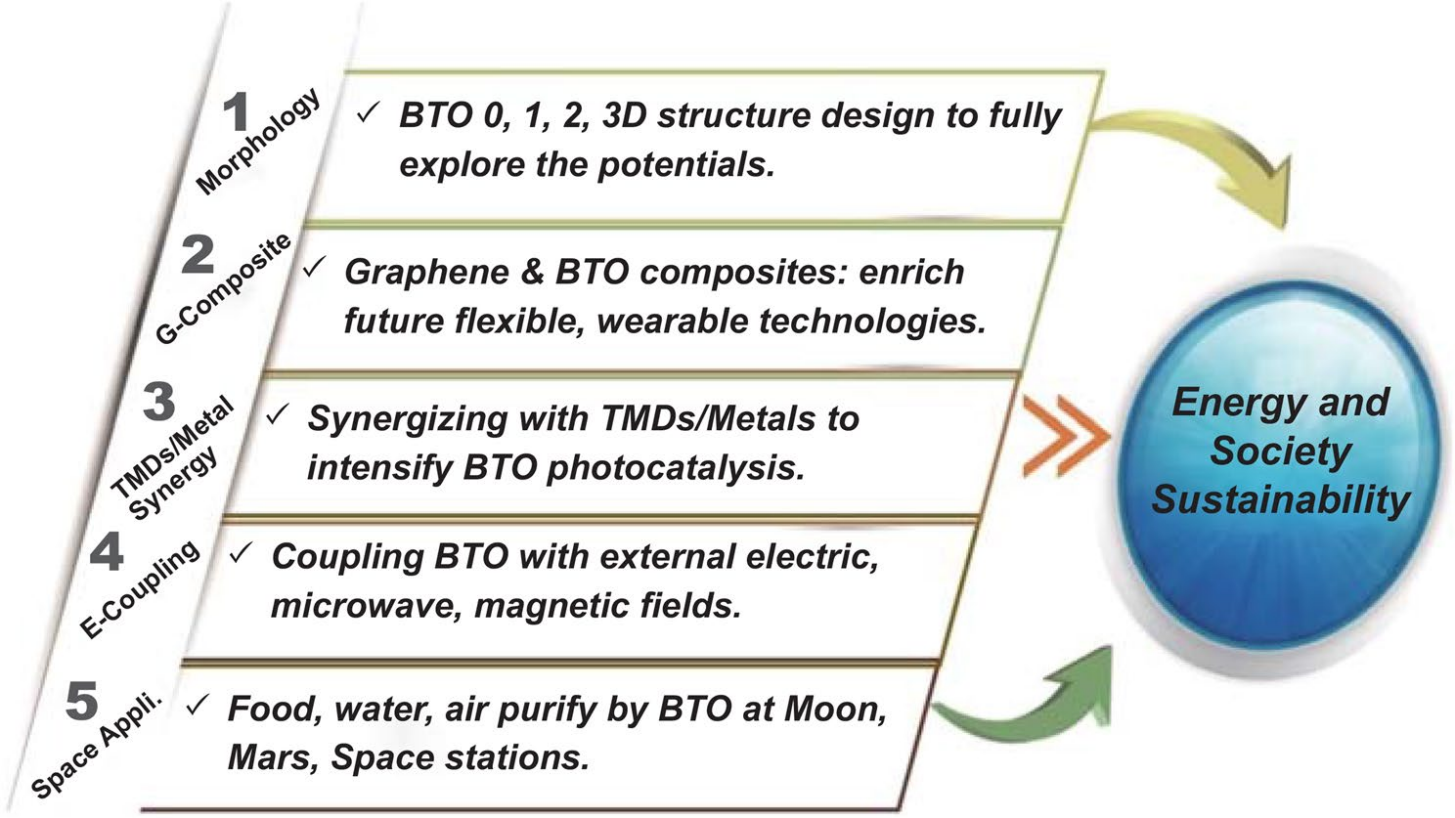
Future research suggestions based on the unique properties of BTO to improve energy and social sustainability

The science and technology exploitation has been speeding up in modern society than any other historical era. Based on the invention of BTO, the research progress towards energy and society sustainability can be promoted from diverse aspects. The forthcoming flourishing research suggestions based on the account of BTO achievements are suggested below (Fig. 5).

- 1. Design and synthesize a BTO specific morphology and structure in a different dimension (0, 1, 2, 3D). Nanostructured materials are essential for photoelectrochemical devices because of their exposed active surfaces, obviously upgraded kinetics, and versatile adaptations [53]. The potential of BTO could be widely explored by investigating it in 0D (quantum dots), 1D (nanowires, nanotubes, nanoribbons, and nanorods), 2D (nanoplates, nanodisks, and nanosheets), and 3D (nanoflowers, nanocoils, and ordered mesoporous framework) forms.
- 2. Construct graphene/carbon composites with BTO for use in flexible and wearable energy devices to advance their mechanical and electron flow properties.
- 3. Synergize BTO with other typical transition metal dichalcogenides and single or dual metal atoms to further boost its photocatalytic performance in terms of yield, selectivity, and long-term stability.
- 4. Couple BTO applications with external fields (electricity, magnetism, plasmonic energy, microwaves, or polarized light). The external fields are expected to influence the photocatalytic process in several ways, such as inducing polarization in reactant molecules (like CO_2_, N_2_, and VOCs) to assist in the dissociation of molecules, prompting chiral molecule pure enantiomer synthesis, and altering the hot carrier migration pathways of the photocatalyst under an electromagnetic wave, so on.
- 5. On the frontier of space science, one of the ultimate goals is to build an environment in which humans could live. Space applications of BTO could lead to a bright future for sustainable human civilization. Currently, the International Space Station is equipped with the “Photocatalytic Oxidation Reactor System” (PORS) for VOC removal during the potable water purification step [54]. And the Kennedy Space Center has developed a visible-light-responsive Ag-doped TiO_2_ catalyst PORS in 2016 for better water purification system [55]. BTO, as an advanced photocatalyst, has shown superior photocatalytic activity than most noble metal-doped TiO_2_ and will enable the efficient acquisition of clean-living necessities (food, water, and air) in the Space living area. Furthermore, researchers have found that up to 10 wt% of TiO_2_ exists in the regional area of Moon’s crust, which can further serve to assist the future human exploration of the Moon [56].

Herein, we have described milestones in TiO_2_ material design, including the development of BTO. Then, we explained our M-EDA phase-selective disordering mechanism and the unique advances offered by BTO in visible-light absorption and exciton disassociation. We continued by discussing applications already achieved and prospective advances from those. Last, we proposed several potential new prospects for BTO that target energy and social sustainability. Relying on the structure specialty and superior accomplishments, the unique NPT-synthesized BTO could offer more socially beneficial applications and approach to commercial, robust visible-light-driven versatile photocatalyst if its potential is fully explored by the research community.

## References

[1] Keidel E (1929). The fading of aniline dyes in the presence of titanium white. Farben-Zeitung.

[2] Fujishima A, Honda K, Kikuchi S (1969). Photochemical reactions of semiconductors. I. Photosensitized electrolytic oxidation on semiconducting n-type TiO2 electrode. Kogyo Kagaku Zasshi.

[3] Fujishima A, Honda K (1972). Electrochemical photolysis of water at a semiconductor electrode. Nature.

[4] Haque F, Daeneke T, Kalantar-Zadeh K, Ou JZ (2018). Two-dimensional transition metal oxide and chalcogenide-based photocatalysts. Nano-micro Lett..

[5] Reyes-Coronado D, Rodriguez-Gattorno G, Espinosa-Pesqueira ME, Cab C, de Coss R (2008). Phase-pure TiO_2_ nanoparticles: anatase, brookite and rutile. Nanotechnology.

[6] Hashimoto K, Irie H, Fujishima A (2005). TiO_2_ photocatalysis: a historical overview and future prospects. Jpn. J. Appl. Phys..

[7] Luttrell T, Halpegamage S, Tao J, Kramer A, Sutter E (2014). Why is anatase a better photocatalyst than rutile?–model studies on epitaxial TiO2 films. Sci. Rep..

[8] West RH, Celnik MS, Inderwildi OR, Kraft M, Beran GJO (2007). Toward a comprehensive model of the synthesis of TiO_2_ particles from TiCl_4_. Angew. Ind. Eng. Chem. Res..

[9] Ngamta S, Boonprakob N, Wetchakun N, Ounnunkad K, Phanichphant S (2013). A facile synthesis of nanocrystalline anatase TiO_2_ from TiOSO_4_ aqueous solution. Mater. Lett..

[10] Qi L, Yu J, Jaroniec M (2011). Preparation and enhanced visible-light photocatalytic H_2_-production activity of CdS-sensitized Pt/TiO_2_ nanosheets with exposed (001) facets. Phys. Chem. Chem. Phys..

[11] Ide Y, Inami N, Hattori H, Saito K, Sohmiya M (2016). Remarkable charge separation and photocatalytic efficiency enhancement through interconnection of TiO_2_ nanoparticles by hydrothermal treatment. Angew. Chem. Int. Ed..

[12] Moztahida M, Lee DS (2020). Photocatalytic degradation of methylene blue with P25/graphene/polyacrylamide hydrogels: optimization using response surface methodology. J. Hazard. Mater..

[13] Ohtani B, Prieto-Mahaney OO, Li D, Abe R (2010). What is degussa (evonik) P25? Crystalline composition analysis, reconstruction from isolated pure particles and photocatalytic activity test. J. Photochem. Photobiol. A: Chem..

[14] Noman MT, Ashraf MA, Ali A (2019). Synthesis and applications of nano-TiO_2_: a review. Environ. Sci. Pollut. Res. Int..

[15] Fujishima A, Zhang X, Tryk D (2008). TiO_2_ photocatalysis and related surface phenomena. Surf. Sci. Rep..

[16] Habibi-Yangjeh A, Asadzadeh-Khaneghah S, Feizpoor S, Rouhi A (2020). Review on heterogeneous photocatalytic disinfection of waterborne, airborne, and foodborne viruses: can we win against pathogenic viruses?. J. Colloid. Interface Sci..

[17] Chen X, Burda C (2008). The electronic origin of the visible-light absorption properties of C-, N- and S-doped TiO_2_ nanomaterials. J. Am. Chem. Soc..

[18] Inturi SNR, Boningari T, Suidan M, Smirniotis PG (2013). Flame aerosol synthesized Cr incorporated TiO_2_ for visible light photodegradation of gas phase acetonitrile. J. Phys. Chem. C.

[19] Ali A, Yassitepe E, Ruzybayev I, Shah SI, Bhatti AS (2012). Improvement of (004) texturing by slow growth of Nd doped TiO_2_ films. J. Appl. Phys..

[20] Chen XB, Liu L, Yu PY, Mao SS (2011). Increasing solar absorption for photocatalysis with black hydrogenated titanium dioxide nanocrystals. Science.

[21] Wang L, Zhang K, Kim JK, Ma M, Veerappan G (2016). An order/disorder/water junction system for highly efficient co-catalyst-free photocatalytic hydrogen generation. Energy. Environ. Sci..

[22] Pan J, Liu G, Lu GQ, Cheng HM (2011). On the true photoreactivity order of 001}, {010}, and {101 facets of anatase TiO2 crystals. Angew. Chem. Int. Ed..

[23] Xu M, Gao Y, Moreno EM, Kunst M, Muhler M (2011). Photocatalytic activity of bulk TiO_2_ anatase and rutile single crystals using infrared absorption spectroscopy. Phys. Rev. Lett..

[24] Hurum DC, Agrios AG, Gray KA, Rajh T, Thurnauer MC (2003). Explaining the enhanced photocatalytic activity of degussa P25 mixed-phase TiO_2_ using EPR. J. Phys. Chem. B.

[25] Ohno T, Sarukawa K, Tokieda K, Matsumura M (2001). Morphology of a TiO_2_ photocatalyst (degussa, P25) consisting of anatase and rutile crystalline phases. J. Catal..

[26] Chen X, Liu L, Huang FQ (2015). Black titanium dioxide (TiO_2_) nanomaterials. Chem. Soc. Rev..

[27] Birch AJ (1944). 117. Reduction by dissolving metals. Part I. J. Chem. Soc. (Resumed)..

[28] Hwang HM, Oh S, Shim J-H, Kim Y-M, Kim A (2019). Phase-selective disordered anatase/ordered rutile interface system for visible-light-driven, metal-free CO_2_ reduction. ACS Appl. Mater. Interfaces.

[29] Oi LE, Choo M-Y, Lee HV, Ong HC, Hamid SBA (2016). Recent advances of titanium dioxide (TiO_2_) for green organic synthesis. RSC Adv..

[30] Pal J, Pal T (2015). Faceted metal and metal oxide nanoparticles: design, fabrication and catalysis. Nanoscale.

[31] Kim Y, Hwang HM, Wang L, Kim I, Yoon Y (2016). Solar-light photocatalytic disinfection using crystalline/amorphous low energy bandgap reduced TiO_2_. Sci. Rep..

[32] Nguyen CTK, Tran NQ, Seo S, Hwang H, Oh S (2020). Highly efficient nanostructured metal-decorated hybrid semiconductors for solar conversion of CO_2_ with almost complete CO selectivity. Mater. Today.

[33] Bak S, Lee SM, Hwang HM, Lee H (2019). Phase-selective modulation of TiO_2_ for visible light-driven charylation: tuning of absorption and adsorptivity. Mol. Catal..

[34] McAllister S, Chen JY, Fernandez-Pello AC (2011). Fundamentals of combustion processes.

[35] Cooke GD, Kennedy RH (2001). Managing drinking water supplies. Lake Reserv. Manag..

[36] Li X, Yu J, Jaroniec M, Chen X (2019). Cocatalysts for selective photoreduction of CO_2_ into solar fuels. Chem. Rev..

[37] da Silva AL, Wu L, Caliman LB, Castro RHR, Navrotsky A (2020). Energetics of CO_2_ and H_2_O adsorption on alkaline earth metal doped TiO_2_. Phys. Chem. Chem. Phys..

[38] Lang X, Chen X, Zhao J (2014). Heterogeneous visible light photocatalysis for selective organic transformations. Chem. Soc. Rev..

[39] Wang L, Xia M, Wang H, Huang K, Qian C (2018). Greening ammonia toward the solar ammonia refinery. Joule.

[40] Xue X, Chen R, Yan C, Zhao P, Hu Y (2019). Review on photocatalytic and electrocatalytic artificial nitrogen fixation for ammonia synthesis at mild conditions: advances, challenges and perspectives. Nano Res..

[41] Schrauzer GN, Guth TD (1977). Photolysis of water and photoreduction of nitrogen on titanium dioxide. J. Am. Chem. Soc..

[42] Stucchi M, Galli F, Bianchi CL, Pirola C, Boffito DC (2018). Simultaneous photodegradation of VOC mixture by TiO_2_ powders. Chemosphere.

[43] Kamal MS, Razzak SA, Hossain MM (2016). Catalytic oxidation of volatile organic compounds (VOCs)—a review. Atmos. Environ..

[44] Li D, Gu AZ, He M, Shi HC, Yang W (2009). UV inactivation and resistance of rotavirus evaluated by integrated cell culture and real-time RT-PCR assay. Water Res..

[45] Sjogren JC, Sierka RA (1994). Inactivation of phage MS_2_ by iron-aided titanium dioxide photocatalysis. Appl. Environ. Microbiol..

[46] Liu M, Sunada K, Hashimoto K, Miyauchi M (2015). Visible-light sensitive Cu(ii)–TiO_2_ with sustained anti-viral activity for efficient indoor environmental remediation. J. Mater. Chem. A.

[47] Nakano R, Hara M, Ishiguro H, Yao Y, Ochiai T (2013). Broad spectrum microbicidal activity of photocatalysis by TiO_2_. Catalysts.

[48] Luo Y, Wang L, Hwang Y, Yu J, Lee J (2021). Binder-free TiO_2_ hydrophilic film covalently coated by microwave treatment. Mater. Chem. Phys..

[49] Yang J, Xiao X, Gong W, Zhao L, Li G (2019). Size-independent fast ion intercalation in two-dimensional titania nanosheets for alkali-metal-ion batteries. Angew. Chem. Int. Ed..

[50] Wang Y, Xue X, Liu P, Wang C, Yi X (2018). Atomic substitution enabled synthesis of vacancy-rich two-dimensional black TiO_2- x_ nanoflakes for high-performance rechargeable magnesium batteries. ACS Nano.

[51] Wang S, Kravchyk KV, Pigeot-Rémy S, Tang W, Krumeich F (2019). Anatase TiO_2_ nanorods as cathode materials for aluminum-ion batteries. ACS Appl. Nano Mater..

[52] Myung S-T, Kikuchi M, Yoon CS, Yashiro H, Kim S-J (2013). Black anatase titania enabling ultra high cycling rates for rechargeable lithium batteries. Energ. Environ. Sci..

[53] Tiwari JN, Tiwari RN, Kim KS (2012). Zero-dimensional, one-dimensional, two-dimensional and three-dimensional nanostructured materials for advanced electrochemical energy devices. Prog. Mater. Sci..

[54] M. P. Nagaraja, Water on the space station. (NASA Science Share the Science, 2000). https://science.nasa.gov/science-news/science-atnasa/2000/ast02nov_1. Accessed 5 Nov 2020

[55] J.L. Coutts, P.E. Hintze, A. Meier, M.G. Shah, R.W. Devor et al., Visible-light-responsive photocatalysis: ag-doped TiO_2_ catalyst development and reactor design testing. 46th International conference on environmental systems. 169 (2016)

[56] Robinson MS, Hapke BW, Garvin JB, Skillman D, Bell JF (2007). High resolution mapping of TiO_2_ abundances on the moon using the hubble space telescope. Geophys. Res. Lett..

